# Pilot study MOVENDOP protocol - impact on quality of life following postoperative osteopathic abdominal mobilizations in patients operated for endometriosis

**DOI:** 10.1371/journal.pone.0323214

**Published:** 2025-05-08

**Authors:** Aurélie Comptour, Caroline Dechambenoit, Fabrice Kwiatkowski, Astrid Vidal, Marie Duval, Marie De Antonio, Aurélie Jacobs, Nicolas Bourdel

**Affiliations:** 1 INSERM, CIC CRECHE Unit, CHU Clermont-Ferrand, Department of Gynecological Surgery, Clermont-Ferrand, France; 2 IFSO, Institut de formation supérieure en ostéopathie, 4 Rue Gallieni, Vichy, France; 3 Université Clermont Auvergne, Blaise Pascal Laboratory of Mathematics, Aubière Cedex, France; 4 CHU Clermont-Ferrand, Biostatistics, Clermont-Ferrand, France; 5 CHU Clermont-Ferrand, Department of Gynecologic Surgery. Clermont Ferrand France; 6 Université Clermont Auvergne, EnCoV, Institut Pascal, UMR CNRS, SIGMA Clermont, Clermont-Ferrand, France; PLOS: Public Library of Science, UNITED KINGDOM OF GREAT BRITAIN AND NORTHERN IRELAND

## Abstract

**Background:**

Surgery remains the gold standard for management of endometriosis, offering significant improvement in patient pelvic pain and quality of life (QoL). Postoperative tissue adhesions can however diminish these benefits, limiting the long-term effectiveness of the intervention. Despite the development of strategies and devices to reduce adhesion formation, their efficacy remains inconclusive.

**Objective:**

This study aims to propose and evaluate a novel approach involving early visceral mobilization and training of patients in abdominal self-mobilization as a means to improve QoL following surgery.

**Methods:**

This pilot study is a prospective, randomized, phase II superiority trial. Patients undergoing surgery for infiltrating endometriosis will be randomized, with a 2:1 ratio, into two groups. The intervention group (n = 42) will receive six sessions of osteopathic visceral mobilization with training in abdominal self-mobilization techniques, one preoperative, and five postoperative during the first month post-surgery. The control group (n = 21) will receive no osteopathic visceral mobilizations but will be offered an osteopathic session after one year. The primary endpoint is a minimum increase of 20 points (on a 100-point scale) in the Endometriosis Health Profile-30 (EHP-30) global score, at one year. Secondary endpoints include assessment of gastrointestinal quality of life (GIQLI), sexual function (FSFI), urinary symptoms (ICIQ-FLUTS), pain catastrophizing (PCS), as well as scar examination, pelvic pain, abdominal flexibility, use of medical and non-medical care, analgesic and hormonal treatments, pregnancy rate, physical activity, sedentary lifestyle and patient compliance. Statistical analyses will be based on a one-sided α=0.05 and β=0.15, assuming a standard deviation of 25 points in the EHP-30 global score. A total of 42 participants in the intervention group and 21 in the control group are required.

**Discussion:**

This trial aims to demonstrate that early and repeated osteopathic sessions following surgery for endometriosis may significantly improve patient QoL.

## Introduction

Endometriosis, characterized by the presence of endometrial-like tissue outside the uterine cavity associated with chronic inflammation, affects approximately 5–10% of women of reproductive age [1–3]. This disease was first described in 1690 and primary symptoms outlined in 1769, but remains a complex and poorly understood disease [4,5]. Endometriosis symptoms are highly heterogeneous, with the most common triad being dysmenorrhea, dyspareunia and chronic pelvic pain. These symptoms can severely impair quality of life (QoL) and contribute to infertility, as previously documented [6–10].

To assess the impact of endometriosis on QoL, several validated scales have been developed. Among these, the generic multidimensional SF-36 questionnaire and the disease-specific EHP-30 scale are the most widely used tools in endometriosis research [11]. Diagnosis and management of endometriosis however, remain challenging, with an average period of 7–8 years between the onset of symptoms and diagnosis, depending on disease severity [12].

Treatment strategies are tailored, based on symptoms, patient age, pregnancy wish, lesion type and comorbidities. Despite frequent use of hormonal therapies as first-line treatment, for refractory patients and certain lesion types, surgery remains the gold standard [13].

A common postoperative complication of gynecological surgery including endometriosis surgery, is peritoneal adhesion formation, which can result in chronic pain, dyspareunia, infertility and gastrointestinal disorders, with significant short- and long-term morbidity [14–16]. Studies report that more than 90% of abdominal surgeries result in adhesions [17–19]. While minimally invasive techniques such as laparoscopy may reduce adhesion formation risk, they cannot entirely prevent adhesion development or recurrence [14,20–24]. Furthermore, endometriosis itself is considered highly adhesiogenic [14,16,25].

During abdominal surgery, a series of events occur at a tissue trauma site, resulting in the formation of adhesions. This process begins with an inflammatory reaction, followed by increased microvascular permeability, the formation of inflammatory exudate, fibrin deposition, invasion by fibroblasts, and ultimately, the development of fibrous tissue connecting the traumatized surfaces [26,27].

To address adhesion formation, various strategies and devices have been introduced, primarily involving physical barriers such as synthetic anti-adhesive membranes, fluid agents, and pharmacological treatments. These barriers act as temporary protective layers over operated surfaces during healing [28,29]. However, their efficacy is largely controversial, with inconsistent outcomes reported in the literature [16,17,21,22,30,31].

Adhesiolysis remains the only treatment for adhesions, but as recurrence following surgical adhesiolysis is common [14,22,23,26], preventing adhesions [15,22,32] relies on quality surgical techniques. Given the high level of morbidity associated with pelvic adhesions, there is a growing need for innovative, non-invasive strategies, such as manual osteopathic visceral mobilizations.

Case reports have suggested that soft-tissue mobilization techniques may provide relief from pain associated with post-surgical adhesions [33–35]. Wassermann’s review reported that immediate application of scar release techniques such as massage, visceral mobilization and myofascial release postoperatively, may prevent ileus, reduce analgesic use and potentially decrease adhesion formation [36]. Wurn et al. also highlighted the role visceral manipulation may play in improving fertility in patients with abdominopelvic adhesions [37].

The primary goals of these mobilization techniques are twofold; firstly to reduce the likelihood of adhesion development through early, repeated and adequate mobilization of postoperative areas, so as to disrupt the formation of adherent tissue, and secondly, to facilitate lymphatic drainage of the abdomen, enabling rapid absorption of intraperitoneal fluids and their reintegration into the circulatory system [38].

While traditional methods such as physical barriers and adhesiolysis, provide limited success, manual osteopathic visceral mobilizations offer a promising alternative. Further research is required to confirm their effectiveness, hone methodologies, and establish evidence-based protocols to alleviate the burden of adhesion-related complications.

We present the protocol for a superiority, prospective, monocentric, randomized, phase II controlled trial with a 2:1 parallel group design. The primary objective of this trial is to evaluate the impact of osteopathic visceral mobilizations on postoperative quality of life in women undergoing surgery for endometriosis. Intervention includes postoperative consultations with an experienced osteopath and training in abdominal self-mobilization, with outcomes assessed at 12 months post-surgery. Secondary objectives include comparing patient quality of life at 6 and 12 months post-surgery, post-operative pelvic pain, scar appearance, abdominal flexibility, range of medical and non-medical care, use of analgesics and hormonal treatments, pregnancy achievement documented at 12 months, physical activity and sedentary lifestyle and patient compliance throughout the study.

This protocol provides a robust framework for investigating the effectiveness of osteopathic interventions in improving postoperative outcomes and quality of life in this patient population.

## Materials and methods

### Setting

The study will be conducted in an academic tertiary referral hospital in Clermont-Ferrand, France which is one of the 5 national expert centers for the management of endometriosis.

This research has obtained the written ethical approval of the French Personal Protection Committee Sud-Est III on July 31, 2024 (2023-A02653-42).

### Study design and participant timeline

**Fig 1:**
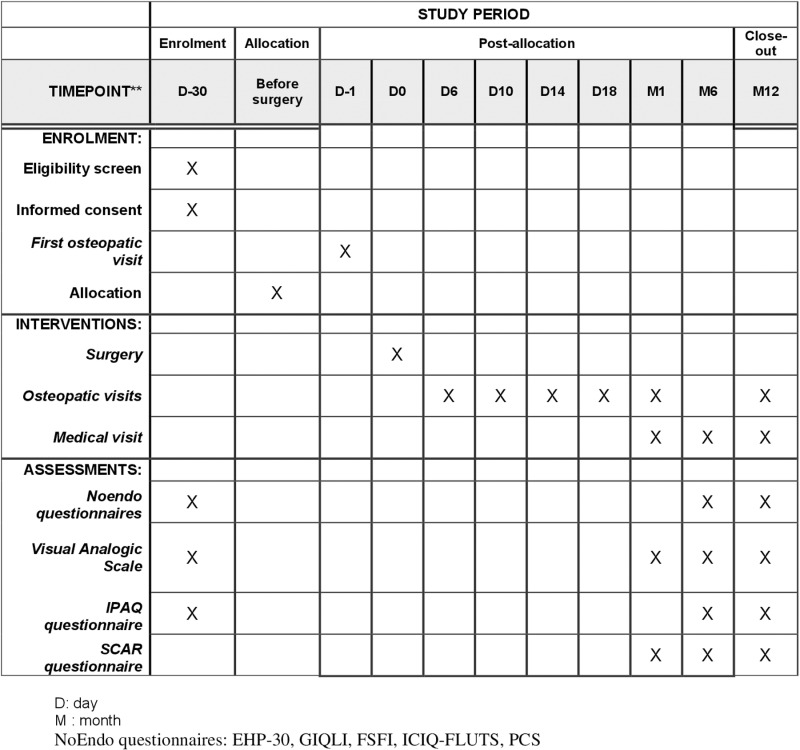
Flowchart of MOVENDOP trial.

**Fig 2:**
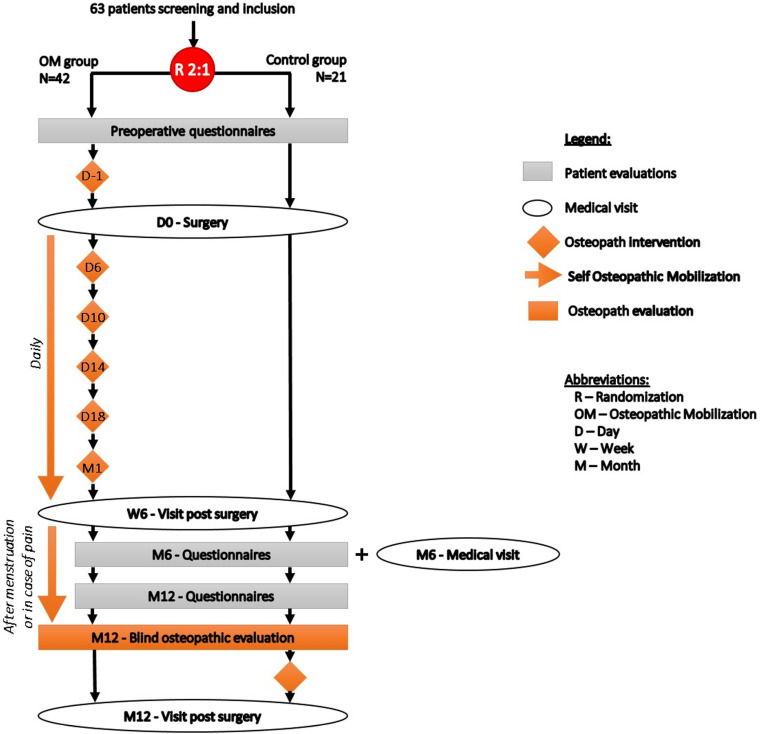
Flow diagram of MOVENDOP trial.

The flow chart of the study is presented in **Fig 1**. This is a superiority monocentric 2:1 randomized parallel group trial comparing reported quality of life, using the 30-item Endometriosis Health Profile (EHP-30) questionnaire, in 2 groups of patients operated for endometriosis. In the experimental group, patients receive pre and post-operative osteopathic consultations. The pre-operative consultation includes learning abdominal self-mobilizations and abdominal diaphragmatic respiration with the osteopath. The 5 post-operative consultations, occurring at 3–5 day intervals, involve visceral mobilizations performed by the osteopath (D6, D10, D14, D18 and W6). In the second group (control group), patients receive no osteopathic mobilizations (**Fig 2**).

Both groups benefit from 3 post-operative consultations with the surgeon (at 6 weeks, 6 and 12 months after surgery) and an osteopathic consultation 1 year after surgery for endpoint assessment (abdominal flexibility). In addition, the control group receives training in visceral self-mobilization. Data from patients, surgeons and osteopaths is collected at baseline (pre-operative visit), surgery, post-operative consultations (6 weeks, 6 and 12 months after surgery), 5 post-operative osteopathic interventions and at 1 year after surgery (**Fig 1** and **Fig 2**).

A follow-up booklet distributed to patients according to their randomization group, will allow patients in the experimental group to provide data concerning abdominal self-mobilizations performed during the first year following surgery (frequency of performing osteopathic maneuvers and evaluation of pain), and for both groups to provide data on abdominal flexibility at 12 months, medical and non-medical treatment received, and analgesic and painkiller use during this period.

### Population

Inclusion criteria are patients aged > 18 requiring surgery for infiltrating endometriosis, who are able to give informed consent for participation in MOVENDOP research and the NoEndo cohort, the French National Observatory for ENDOmetriosis developed by Clermont-Ferrand University Hospital (ethical approval 2022-CF037), data being mostly collected via the NoEndo digital platform.

Non-inclusion criteria include patients with an indication for surgery for superficial endometriosis, patients under guardianship, trusteeship or legal protection, pregnant or breastfeeding patients, patients non- affiliated to the French social security system, non-French speakers, refusal to participate or patients participating in another study.

### Recruitment and consent

Patients will be recruited during the pre-operative consultation by one of the surgeons involved in this study, and receive information on research objectives and procedures. All patients will benefit from a minimum seven-day reflection period. The second consultation with the surgeon is dedicated to providing further information and the signing of consent forms. Based on the number of surgeries for infiltrating endometriosis performed in our service, the rate of inclusion is estimated at 3 patients per month. The period of participation by patient is one-year post-surgery.

### Blinding

For both groups, osteopathic consultation at 1-year post-surgery is carried out by an independent osteopath, blind to patient group allocation. Abdominal tissue flexibility will be evaluated for all participants at this consultation.

### Intervention

#### Surgical technique.

Our surgical technique has previously been described [8,39]. Endometriosis staging is performed after surgery based on rAFS scores. Using laparoscopy as an exclusive approach, complete surgical treatment of endometriosis will be performed, which allows accurate disease stage classification. Superficial peritoneal lesions will be treated using bipolar coagulation or peritoneal excision, and endometriomas by cystectomy as described previously [40]. Recto-vaginal nodules will be treated by shaving or by colorectal resection should a shaving procedure be deemed unsatisfactory. This surgical technique has been previously detailed [41]. In cases of endometriosis invading the sigmoid colon or the small bowel, segmental resection will be performed and ureteral involvement treated by ureteroneocystostomy where necessary, as previously described [42]. In the event of bladder lesions invading the mucosa, a partial cystectomy will be performed and closed using single- or double-layer suturing.

Post-operative consultations with the surgeon (at 6 weeks, 6 and & 12 months) will allow assessment of pain, scarring and post-operative complications.

#### Osteopathic interventions.

Patients in the experimental group will receive training in abdominal self-mobilizations at an initial pre-operative consultation, to ensure they can perform these techniques independently and take an active role in their treatment. While some maneuvers remain managed by the osteopath, patients are required to perform frequent sessions of self-mobilization of the abdomen, combined with Abdominal Diaphragmatic (AD) breathing. AD breathing, the self-mobilization procedure and osteopathic intervention are detailed in appendix 1 and 2.

Patient participation allows:

- Optimization of treatment provided by the osteopath, who cannot be seen every day
- Pain management using the simple tools available to patients
- Association of this part of the body with sensations other than pain
- Patients to take an active role in their treatment.

Patients in the intervention group receive six sessions with the osteopath.

**Session 1** Pre-surgical session focusing on the teaching of self-mobilization **(****Appendix 1**)

- AD breathing in 6 steps
- Abdominal self-mobilization of:
- Three lower abdominal regions: right iliac fossa, hypogastrium and left iliac fossa
- Abdominal scars
- Sequence of maneuvers and repetitions:
- Ten AD breaths
- Abdominal self-mobilizations (of the 3 regions + scars)
- 10 AD breaths

The procedure is to be repeated 3 times a day during the first month.

In the first postoperative week if self-mobilizations are difficult to perform (e.g., high pain levels or apprehension), the patient will perform AD breathing more frequently, namely 6 cycles of 10 AD breaths per day.

**Sessions 2–6** (D6, D10, D14, D18 and W6): Visceral mobilizations by the osteopath are performed during the month following surgery, with sessions at 3–5-day intervals (Appendix 2, **Fig 1** and **Fig 2**).

During these consultations, the osteopath ensures self-mobilizations are being correctly carried out, readjusts if necessary and answers any patients questions. The osteopath also checks that patient follow-up notebooks are completed.

These sessions also involve:

a/ Achieving a balanced diaphragmatic range
b/ General abdominal mobilizations: hypogastric zones and right and left iliac fossae
c/ Abdominal scar manipulation
d/ Maneuver stages:
  1. Ten AD breaths
  2. Work on achieving balance in diaphragmatic range
  3. General abdominal mobilizations
  4. Ten AD breaths

After the first postoperative month, the patient performs the self-mobilizations on a daily basis during the week following menstruation. In patients whose cycle is blocked by contraception, the procedures are recommended as analgesic in the event of abdominal pain.

Explanations and demonstrations given by the osteopath ensure correct understanding and sequence performance by the patient.

Patients receive a summary document and access to an online video and are requested to note the frequency and periodicity of the sequences performed, on an online document.

**Session at one-year** All patients in both groups will be seen one year after surgery for an osteopathic consultation with a dual aim:

a/ Evaluation of abdominal flexibility

Assessment of abdominal flexibility is performed by the osteopath using the mobilizations of the hypogastric regions, right iliac fossa and left iliac fossa as performed during previous consultations.

Abdominal flexibility is scored by the osteopath using a visual analogue scale (VAS) ranging from 0 = maximal mobility to 10 = absence of mobility, based on the osteopath’s experience of movement in these areas. Differences in perceived amplitudes are scored for each axis of movement (up/down, in/out and clockwise/counterclockwise rotation).

In cases of pain, a second VAS is used for assessment, indicating the area and direction of the mobilization that triggered the pain, or by noting the pain score as null.

b/ Self-mobilization training for control group patients:

Patients are taught self-mobilizations and the 6 cycles of AD breathing by an osteopath. They also learn how these exercises contribute to management of abdominal pain and prevention of adhesions associated with endometriosis relapse.

### Outcomes

The primary outcome is the percentage variation in baseline EHP30 questionnaire scores between the pre-operative visit and at 1 year after surgery.

To measure QoL, the authors chose the EHP-30 questionnaire, one of the most frequently used scales for quantification of perceived health, specific to endometriosis [11].

The EHP-30 contains 30 items ranging from 0 (best health status) to 100 (worst health status). The core questionnaire items are grouped into 5 main scales: pain (11 items), control and powerlessness (6 items), emotional well-being (6 items), social support (4 items) and self-image (3 items). A modular questionnaire, that may not apply to all women with endometriosis, comprises 23 items grouped into six scales: ‘Work’ (5 items), ‘Relationship with children’ (2 items), ‘Sexual intercourse’ (5 items), ‘Medical profession’ (4 items), ‘Treatment’ (3 items), and ‘Infertility’ (4 items) [7].

Secondary outcomes are listed below:

1/
  - a/ The following validated questionnaire scores: EHP-30, GIQLI (digestive function), FSFI (sexual function), ICIQ-FLUTS (urinary tract symptoms) and PCS (pain catastrophizing) evaluated at the pre-operative visit, and at 6 and 12 months.
  - b/ VAS of pelvic pain reported at the pre-operative visit, then post-operatively at H6, D1, 1 month, 6 and 12 months and expressed as a percentage variation compared with pre-operative visit results.
  -c/ Post-operative examination of scar appearance by the surgeon at 6 and 12 months using the SCAR (Scar Cosmesis Assessment and Rating) scale.
2/
  - a/ VAS of abdominal flexibility in the following areas: hypogastrium, and right and left iliac fossa, at one year assessed by an independent osteopath, scores ranging from 0 to 10 (0 corresponding to normal flexibility and 10 to total rigidity).
  - b/ Consultations in addition to conventional care (medical and non-medical)
  - c) Use of analgesics and hormonal treatments reported by patients during the first postoperative year
  -d) Pregnancy achievement during the first postoperative year
3/ The International Physical Activity Questionnaire (IPAQ) assessed before surgery, at 6 and 12 months after surgery.
4/ Short-term compliance in terms of number of days and frequency of mobilizations, assessed on a weekly basis for up to 6 weeks,. Long-term compliance is assessed monthly post-cycle in terms of whether and how often mobilizations were performed, and if used in cases of pain.

### Sample size

The required number of subjects is based on a comparison between the two randomized groups (experimental group *vs*. control) of the percentage variation in EHP-30 questionnaire scores between the pre-operative visit and at 1 year, with a two-sided first-species risk of error of 5%. For ethical reasons, we chose a 2:1 ratio for randomization.

In the literature, Goncalves et al. reported on the evolution of EHP30 scores (before/after) for a control and treatment group receiving an intervention (yoga sessions) [43]. Variations were small in the control group for the 5 main EHP-30 dimensions, and ranged from 20 to 35 points for the treatment group. Pokrzywinski et al [44]. showed the same before/after differences for a control and treatment group (Elagolix molecule), and defined thresholds of between 20 and 35 points for clinically significant differences.

To demonstrate a difference of 20 points associated with a standard deviation of 25, in percentage variation for EHP-30 questionnaire scores between the pre-operative visit and 1 year, with a statistical power greater than 80%, 38 patients are required in the experimental group and 19 in the control group. To allow for patients leaving the protocol and loss to follow-up, 10% more patients are required in each group. Thus, a total of 63 patients will be recruited (42 in the experimental group and 21 in the control group).

### Randomization

Patients will be randomly assigned to the two groups using REDCap® software, with a 2:1 ratio.

### Data collection methods

Data will be collected using 2 different tools: REDCap® electronic data capture and the NoEndo platform. An eCRF (electronic Case Report Form) using REDCap® will be used by the surgeon and clinical research associates to collect patient clinical data at each visit, as per the protocol, i.e., demographic and relevant background data, surgical indication, scar examination, pain assessment, postoperative complications, sick leave, hormonal treatment and follow-up data during the 1^st^ year following surgery for each group. This data concerns abdominal self-mobilizations for the experimental group, and abdominal flexibility and extent of medical and non-medical treatment for both groups. Quality-of-life data will be measured using questionnaires specific to endometriosis (EHP-30, GIQLI, FSFI, ICIQ-FLUTS and PCS), which patients will complete via the platform NoEndo. Comparison of the different scores obtained pre-operatively and at 6 and 12 months post-operatively will enable analysis of changes in quality of life.

### Data management

#### Data quality.

Study data will be collected and managed using REDCap electronic data capture tools (version 13.1.28., Vanderbilt University) hosted at Clermont-Ferrard UHC [45,46]. REDCap (Research Electronic Data Capture) is a secure, web-based software platform designed to support data capture for research studies, providing 1) an intuitive interface for validated data capture; 2) audit trails for tracking data manipulation and export procedures; 3) automated export procedures for seamless data downloads to common statistical packages; and 4) procedures for data integration and interoperability with external sources. Data entry is secured by encryption and a login and password for each user. Data will be collected when obtained and an explanation provided for any missing data.

The NoEndo ISO certified platform provides a double authentication system for patients and health professionals, accessible via the Internet and an application (available for android). To avoid missing data, a number of key items are mandatory, preventing patients from saving an entry page should an answer be missing. Coherence testing and outlier detection implemented on the platform ensures data quality and avoidance of outliers.

All data will be monitored according to specifications defined for this type of study, with special attention paid to limiting missing data.

#### Data privacy.

All subject data collected and transmitted to the sponsor by the investigators (or any other specialist) will be rendered anonymous.

Only the first letter of the subject’s first name and surname will be recorded, together with a specific coded number to indicate the order of subject inclusion.

The sponsor will ensure that all subjects taking part in the research provide written consent for access to individual data, and that strictly necessary for research quality control.

On REDCap® and NoEndo, an anonymous number will be assigned to each patient. For database interoperability (eCRF REDCap® and NoEndo) and to guarantee data confidentiality, NoEndo anonymous numbers will be communicated to REDCap®.

### Statistical methods

Statistical analyses will be carried out using R software (R Core Team (2022). R: A language and environment for statistical computing. R Foundation for Statistical Computing, Vienna, Austria. URL https://www.R-project.org/.).

Descriptions are given in number (N) and percentage (%) for qualitative variables, and in mean and standard deviation (SD) or median and interquartile range for quantitative variables, according to their distributions (Gaussian or not). Missing data will be quantified. Normality will be analyzed using the Shapiro-Wilk test and histograms.

Patients will be described and compared between groups at inclusion according to compliance with eligibility criteria, epidemiological characteristics and clinical features. A description of protocol deviations, patients allocated according to these deviations and reasons for drop-out will also be provided.

Initial comparability of the two groups will be assessed on the main patient characteristics and potential factors associated with the primary endpoint. Any difference between the two groups relating to these characteristics will be determined on the basis of clinical and statistical considerations.

Comparisons between groups will be made systematically 1) without adjustment 2) by adjusting for those factors for which distribution may be unbalanced between groups despite randomization, such as contraception, level of disease severity and post-operative complications. All statistical tests will be performed using a two-sided alpha level of 5%. Certain analyses involving secondary endpoints will be mainly exploratory in nature. As discussed by Feise et al. [47] adjustment of alpha risk will not be proposed systematically, but on a case-by-case basis in the light of clinical and statistical considerations.

The main analysis will be carried out on an intention-to-treat basis. An intention-to-treat population refers to all randomized patients except those for whom consent is not obtained or who withdraw their consent concerning use of their data. A per-protocol analysis will be considered of all randomized patients except for those with one or more major deviations defined as: patients ineligibility, uncompliant patients in the experimental group (less than once a day on average over the first post-operative month), or control group patients who perform visceral mobilizations more than 4 times during the year of their own initiative.

The main analysis will be based on a comparison between the experimental and control groups using a Student’s t test or a Wilcoxon test according to distribution (Gaussian or not). Results will be expressed in terms of effect size and associated 95% confidence intervals.

The main analysis will be supplemented by a multivariate analysis of the linear regression type, to take into account covariates selected on the basis of univariate analysis results and their clinical relevance, including age, contraception and previous use of osteopathic mobilization. If necessary, the dependent variable under study may be modified. Particular attention will be paid to the study of multicollinearity (Farrar and Glauber test and VIF indicator) and to the conditions of the model application, notably the normality of residuals. Results will be expressed in terms of regression coefficients and 95% confidence intervals. Subgroup analyses will be proposed for the primary endpoint. The group x subgroup interaction, derived from the linear model described for the main multivariate analysis, will be analyzed and the result expressed in terms of associated regression coefficients and 95% confidence intervals.

Changes in quantitative evaluation criteria (secondary objective 1) based on longitudinal data (measurements repeated over time) will be studied using linear mixed models. These models take into account the correlation of measurements for the same patient. The patient will therefore be considered as a random effect in the model. Time will be considered as both a fixed and a random effect, with dependent variable transformation if necessary. Multivariate models will be considered. The comparison between the two randomization groups will be evaluated by adding the group and its interaction with time as fixed effects to the model. Models can be adjusted for age and contraception, and variable selection can be performed. Results will be expressed in terms of regression coefficients and 95% confidence intervals. Similarly, developments in qualitative evaluation criteria (secondary objective 4) will be based on generalized mixed models of the logistic type. Results will be expressed in terms of odds ratio and 95% confidence intervals.

Comparisons at 1 year will be made using standard statistical tests visit (secondary objective 2). To investigate the dependence between two qualitative variables (including randomization group and fertility), the Chi2 test or Fisher’s exact test (depending on subject numbers) will be applied to a qualitative variable. To compare a quantitative variable (including abdominal flexibility) with a two-modality qualitative variable (including randomization group), a t-test or a Wilcoxon test will be applied, depending on distribution (Gaussian or not).

To identify patient profiles based on the sub-domains of quality of life assessed during the pre-operative visit (secondary objective 3), a factorial analysis will be carried out, followed by a classification method. A Principal Component Analysis (PCA) will be used for further detailed analysis of quantitative data. A hierarchical ascending classification (HAC), based on the results of the factorial analysis will then be carried out. To compare these different groups in terms of improvement in post-operative quality of life, analysis will be based on a comparison using a Student’s t test or a Wilcoxon test according to distribution (Gaussian or not) if classification results in a typology of 2 groups, or using an ANOVA or a Kruskal-Wallis (according to distribution) if classification results in a typology of more than 2 groups.

### Status and timeline

The first patient was recruited on January 27, 2025. A 2-year recruitment period is planned, patient recruitment will therefore be completed in January 2027. The patient follow-up period is 1 year, so the end of data collection is scheduled for January 2028. The results of the study are expected during the year 2028.

## Discussion

This trial investigates a new approach for patients undergoing surgery for endometriosis, which aims to improve patient quality of life and prevent peritoneal adhesion formation. To this end patients receive repeated visceral osteopathic mobilizations immediately after surgery, followed by training in abdominal self-mobilizations. To our knowledge, no randomized controlled trial of rigorous methodology has as yet focused on evaluating the efficacy of osteopathic manipulative treatment in post-operative follow-up of endometriosis.

Currently, there is little published data on visceral mobilizations in humans, and such studies are generally non-randomized, involve small numbers of patients and lack scientific and methodological quality. Bove et al published pre-clinical data focusing on the role of visceral mobilization in the prevention and treatment of peritoneal adhesion in rats [48]. Several case studies have been published suggesting the role of soft tissues mobilization in pain reduction in humans, following post-surgical adhesion formation [33–35]. Wasserman et al.’s review reported on only two related randomized clinical trials [36]. The first trial highlighted use of the Cellu M50 device (LPG, Valence, France) for mechanical massage of the abdominal wall for pain reduction following colectomy [49]. The second reported use of pelvic and abdominal diaphragm myofascial release, direct scar release, skin roll and lumbo-thoracic massage for reducing chronic pain following cesarean section [50].

While use of such therapies have been shown to lead to positive results in other indications (oncology, thoracobrachial outlet syndrome, reticulopathy) [51,52], current data in the literature concerning osteopathic interventions in endometriosis patients remain poor, with no randomized trial involving an adequate number of patients.

Daraï et al. performed a prospective pilot study to evaluate the impact of osteopathic manipulative treatment on quality of life in patients with deep infiltrating endometriosis and reported a significant improvement in several SF36 quality of life questionnaire items in 12 of the 15 patients who received osteopathic manipulations [53]. This study however focused uniquely on patients with colorectal involvement, which corresponds to only 5–12% of patients with deep infiltrating endometriosis. Furthermore, the number of osteopathic manipulations performed was not provided and the median time between pre and post SF36 evaluation was 24 days, a notably short study period. Daraï et al. also conducted a prospective study in 46 patients with colorectal endometriosis, and reported on symptoms and quality of life after osteopathic manipulative therapy [54]. The findings showed a significant improvement in physical and psychological SF36 items in 77% (p < 0.001) of patients following 28 days of therapy. Patients who had undergone surgery for deep endometriosis were however excluded and no details were provided on the number of osteopathic manipulations performed over the 28-day evaluation period.

Although both studies report improved quality of life in patients following osteopathic manipulation, they concerna small number of subjects, cover a very limited period of time and involve a specific case of endometriosis with colorectal involvement without previous surgery. To date, no study has investigated the effect of osteopathic manipulation on post-operative adhesions following endometriosis surgery.

Osteopathy is an alternative medicine essential for management of endometriosis. These patients require multidisciplinary management involving medical specialists (surgeons, pain physicians, gynecologists), supportive care (physical activity, psychological support) and alternative medicine (osteopathy, acupuncture, yoga, sophrology, phytotherapy, etc.) [13,43,55,56].

The major strengths of our study are that it is a prospective randomized study, that includes all patients operated for deep endometriosis, who receive repeated osteopathic manipulations performed over a long period, and a final evaluation at 1 year. Furthermore, our treatment involves the patient playing an active role in their care. The patient receives training in self-mobilization techniques that can bring pain relief and is provided with a follow-up booklet for reporting on pain and well-being.

As there is no method for quantifying adhesions outside of surgery, the primary objective and endpoint of this study were difficult to determine. In accordance with the literature, the assumption was made that adhesions would diminish with improvement in visceral mobility, quality of life and pain reduction [33].

A monocentric design may be considered a weakness of our study. However, a local pilot study presents cost benefits and allows consistent collaboration with an osteopath experienced in treating our patient group. A multicenter trial would require prior therapist training, to ensure a standardized osteopathic approach.

If the expected results are observed in this pilot study, a subsequent large-scale prospective study would allow investigation of therapeutic solutions that favor reduction in adhesions in post-operative endometriosis patients.

## Supporting information

Appendix 1 techniques performed by the patient. (DOCX)

Appendix 2 techniques performed by the osteopath. (DOCX)
