# Supplementary material for: Pilot study MOVENDOP protocol - impact on quality of life following postoperative osteopathic abdominal mobilizations in patients operated for endometriosis
Source: PLoS One. 2025 May 8;20(5):e0323214. doi: 10.1371/journal.pone.0323214 (PMC12061089; doi:10.1371/journal.pone.0323214)
Supplement: Appendix 1 — (DOCX) [file pone.0323214.s001.docx]

**Appendix 1: techniques performed by the patient**

a/ 6- step AD breathing

The patient lies on her back and positions a large cushion under her knees, another under her posterior (providing a slope which relieves pressure exerted by the abdominal viscera on the viscera of the small pelvis, and favors liquid drainage in the lymphatic channels)

The six stages for AD breathing

• The patient begins with an abdominal inspiration, i.e. inflates the belly

• Then continues with a thoracic inspiration, by inflating the thorax

• Next there is an apnea stage, lungs full, of approximately 6 seconds

• The patient exhales, first abdominally, actively (by contracting both the abdominal and perineum)

• Then at the thoracic level by descending the ribs (maintaining abdominal/perineal contraction),

• The AD breath ends with an expiratory apnea of approximately 6 seconds.

b/ Abdominal self-mobilizations

• Mobilization of the 3 lower abdominal regions; left iliac fossa, hypogastrium and right iliac fossa

The patient puts one hand in the other to form a cup and positions hands in the fold of the groin (to mobilize the iliac fossa regions) or above the pubis (to mobilize the hypogastric region) by directing the tips of the fingers towards the bottom and lowest points of each zone.

With the hands positioned, the patient leans her bust forward, until forearms are on her thighs. The abdomen is relaxed and positioned over the hands.

The patient then flexes her hands, making contact with the visceral mass, and exerts a slight traction towards the umbilicus, until intra-abdominal mobilization is felt, taking care to remain below the pain threshold.

Once intra-abdominal mobilization is felt, the patient can relax the flexion of the hands. The process “mobilization/relaxation” is then repeated 10 times, after which the patients stands, shifts her hands to the neighboring area and starts again until all three zones have been mobilized.

• Mobilization of abdominal scars

The patient lies down on her back with a cushion under the knees, another under her posterior. Taking the abdominal scars (entry point for laparoscopic instruments) between the thumb and index fingers, the scars are moved in a circular manner (5 times clockwise, 5 times anti-clockwise).
