# Supplementary material for: Pilot study MOVENDOP protocol - impact on quality of life following postoperative osteopathic abdominal mobilizations in patients operated for endometriosis
Source: PLoS One. 2025 May 8;20(5):e0323214. doi: 10.1371/journal.pone.0323214 (PMC12061089; doi:10.1371/journal.pone.0323214)
Supplement: Appendix 2 — (DOCX) [file pone.0323214.s002.docx]

**Appendix 2: techniques performed by the osteopath**

a/ Maintaining diaphragmatic movement

To achieve the best possible contraction, a muscle at rest must have a balanced state of tension with respect to its internal and external movements.

The patient lies on her back, with a large cushion under the knees (to facilitate relaxation of the abdominal wall).

Place thumbs under the lower costal edge, opposite the insertions of the diaphragm, and release fingers from the lower ribs.

Ask the patient to breathe in deeply and accompany the expansion of the lower ribs. At the end of the inspiration, passively allow further expansion, (thus bringing the diaphragm further on itsdownward track). Maintain the position during passive expiration, and repeat the process for 3 successive breathing cycles.

Release the tension, without modifying the position of the hands.

Next, request active exhalation (i.e. contraction of both the abdominal/perineal muscles) and accompany compression of the lower ribs. As the exhaling process completes, passively allow further compression (the diaphragm continues on its upward track). Maintain the position during inspiration, and repeat for 3 successive breathing cycles.

b/ General abdominal mobilizations

The patient has 3 different positions:

• 1/ lying on her back, with a large cushion under the knees (to promote relaxation of the abdominal wall)

• 2/ lying on the left side, then right (knees bent and superimposed)

• 3/ standing, bust forward and leaning on the table, head resting on crossed forearms, lower limbs pelvis width apart (half-procubitus position)

Alternating these positions allows improved and targeted relaxation of each mobilized zone, i.e. the hypogastric, right iliac fossa and left iliac fossa areas.

The hands of the osteopath “spoon” the visceral content of each area from below, initially the hands are positioned along the fold of the groin (for iliac fossae) and along the upper edge of the pubis (for the hypogastric zone). Contact is made by the hypothenar edge of the hand, with sufficient downward pressure to access the level of the organs (i.e. beyond the abdominal wall). Mobilization involves the 3 planes: up/down, inside/outside, clockwise/anti-clockwise rotation, with speed of actions and range of motion such as to keep below the patient’s pain threshold.

Approximately fifteen movements are performed per zone.

Some areas may be mobilized more than others, depending on the surgical procedure e.g. release of the left ureter requires repeated mobilization of the left iliac fossa.

c/ Abdominal scars

Approximately fifteen multidirectional mobilizations are performed, depending on the cicatrization progress and related pain.
